# Effect of RNAi-mediated silencing of the TaAOS2 gene on phytohormone accumulation, growth and productivity in bread wheat

**DOI:** 10.18699/vjgb-25-124

**Published:** 2025-12

**Authors:** D.N. Miroshnichenko, A.V. Pigolev, E.A. Degtyaryov, V.I. Degtyaryova, V.V. Alekseeva, A.S. Pushin, S.V. Dolgov, T.V. Savchenko

**Affiliations:** Institute of Basic Biological Problems of the Russian Academy of Sciences, Pushchino, Moscow region, Russia Branch of Shemyakin–Ovchinnikov Institute of Bioorganic Chemistry of the Russian Academy of Sciences, Pushchino, Moscow region, Russia; Institute of Basic Biological Problems of the Russian Academy of Sciences, Pushchino, Moscow region, Russia; Institute of Basic Biological Problems of the Russian Academy of Sciences, Pushchino, Moscow region, Russia; Institute of Basic Biological Problems of the Russian Academy of Sciences, Pushchino, Moscow region, Russia Branch of Shemyakin–Ovchinnikov Institute of Bioorganic Chemistry of the Russian Academy of Sciences, Pushchino, Moscow region, Russia Pushchino Branch of Russian Biotechnological University, Pushchino, Russia; Branch of Shemyakin–Ovchinnikov Institute of Bioorganic Chemistry of the Russian Academy of Sciences, Pushchino, Moscow region, Russia; Branch of Shemyakin–Ovchinnikov Institute of Bioorganic Chemistry of the Russian Academy of Sciences, Pushchino, Moscow region, Russia; Branch of Shemyakin–Ovchinnikov Institute of Bioorganic Chemistry of the Russian Academy of Sciences, Pushchino, Moscow region, Russia; Institute of Basic Biological Problems of the Russian Academy of Sciences, Pushchino, Moscow region, Russia

**Keywords:** allene oxide synthase, jasmonates, Triticum aestivum L., RNA interference, wounding, алленоксидсинтаза, жасмонаты, Triticum aestivum L., РНК интерференция, поранение

## Abstract

Reverse genetics methods are actively used in plant biology to study the functions of specific genes responsible for the adaptation of plants to various environmental stresses. The present study describes the production and primary characterization of transgenic bread wheat with silenced expression of allen oxide synthase (AOS). AOS is a key enzyme involved in the initial step of biosynthesis of stress-related phytohormones known as jasmonates. To induce silencing of AOS in wheat, we designed the RNA interference (RNAi) vector containing an inverted repeat region of the TaAOS2 gene cloned from genome DNA of cv. Chinese Spring. With the help of biolistic-mediated transformation, a number of transgenic Chinese Spring plants have been produced. Real-Time PCR analysis confirmed the suppression of target gene expression, since transgenic dsRNAi lines accumulated only 21–44 % mRNA of TaAOS2 after leaf wounding compared to the wound-induced level in non-transgenic control. Gas chromatography–mass spectrometry revealed that the silencing of TaAOS2 substantially reduced the accumulation of jasmonic acid (JA) and jasmonoyl-isoleucine conjugate (JA-Ile), while the production of other phytohormones, such as abscisic acid and salicylic acid, was not affected. TaAOS2-silenced lines were characterized by shorter leaves at the juvenile stage, demonstrated a tendency towards reduced plant height and decreased grain weight, while the average flowering time and plant fertility (number of seeds per spike) were not affected. The obtained transgenic lines in combination with AOS-overexpressing lines can be used for further detailed analysis of the adaptive responses controlled by the jasmonate hormonal system.

## Abstract (ru)

Методы обратной генетики активно используются в биологии растений для изучения функций генов, ответственных за адаптацию растений к различным стрессовым факторам окружающей среды. Данная работа посвящена получению и первичному анализу трансгенных растений мягкой пшеницы с подавленной экспрессией одного из генов алленоксидсинтазы (АОS). АОS является ключевым ферментом, участвующим в начальном этапе биосинтеза стрессовых фитогормонов, известных как жасмонаты. Для подавления экспрессии гена АОS в пшенице создан вектор для РНК-интерференционного замалчивания, содержащий инвертированную область TaAOS2 из генома яровой мягкой пшеницы сорта Chinese Spring. Путем биобаллистической генетической трансформации получен ряд трансгенных растений Chinese Spring, в которых подавление экспрессии целевого гена подтверждено с помощью количественной ПЦР в режиме реального времени, а изменение уровней накопления отдельных жасмонатов выявлено методом газохромато- масс-спектрометрии. В стрессовых условиях (механическое поранение листовой пластинки молодых растений) трансгенные dsRNAi линии накапливали лишь 21–44 % мРНК TaAOS2 по сравнению с нетрансгенным пораненным контролем пшеницы. Подавление экспрессии гена TaAOS2 существенно снизило накопление жасмоновой кислоты и конъюгата жасмоноил-изолейцина, в то время как уровень содержания в листьях других фитогормонов, таких как абсцизовая кислота и салициловая кислота, не изменился. Трансгенные линии пшеницы с подавленной экспрессией гена TaAOS2 демонстрировали уменьшение длины листьев на ранних стадиях развития растений; также для них была характерна тенденция к сокращению высоты растений и снижению массы зерна. При этом замалчивание не привело к изменению сроков цветения и не повлияло на фертильность растений, так как среднее количество семян в колосе не изменилось. Полученные трансгенные линии пшеницы в сочетании с линиями, сверхэкспрессирующими АОS, служат хорошим инструментом для дальнейшего детального анализа адаптивных реакций растений, контролируемых жасмонатным сигналингом.

## Introduction

Allen oxide synthase (AOS) is one of the key enzymes in the biosynthesis pathway of jasmonic acid (JA) and its derivatives, lipid-derived plant phytohormones collectively called jasmonates. Jasmonates have various biological functions, including the regulation of developmental and aging processes, activation of the defence system under abiotic stresses, and triggering the immune response upon attacks by fungal pathogens and herbivores. In brief, the role of AOS in the JA biosynthesis pathway is the production of a short-lived intermediate that is converted to various non-volatile oxylipins in plant cells. AOS is a cytochrome P450 enzyme that is localized in chloroplasts and acts as a hydroperoxide dehydratase, converting fatty acid hydroperoxides into allene oxides. Allene oxide synthase is generally divided into three categories depending on the specific catalytic substrate: 13-AOS with activity to 13-hydroperoxy-9,11,15-octadecatrienoic acid (13-HPOT), 9-AOS with activity to 9-hydroperoxy-9,11,15-octadecatrienoic acid (9-HPOT), and 9/13-AOS with a mixed activity to both 9-HPOT and 13-HPOT (Jiang et al., 2009). Allene oxide formed by 13-AOS is rapidly cyclized with the help of allene oxide cyclase to a more stable product, 12-oxo-phytodienoic acid (OPDA), the biologically active precursor of JA.

Due to the involvement of AOS-derived oxylipins in signalling and coordination of diverse adaptive responses, AOS encoding genes have been the focus of researchers’ attention for more than 30 years. To date, AOS genes have been identified in different dicot and monocot species; the number of AOS genes varies from a single copy in Arabidopsis (Laudert et al., 1996) to a family of 36 genes in the genome of sugarcane (Saccharum officinarum) (Sun et al., 2023). The AOS gene family is relatively conserved in structure, but expression pattern analysis demonstrated that different homologues were involved in various, sometimes distinct, defensive responses across different plant species. In Arabidopsis, the model species for the study of the functioning of the jasmonate system for the last decades, the expression of AOS is upregulated by a virulent fungal pathogen, mechanical stress, and the application of jasmonates and salicylic acid (SA) (Laudert, Weiler, 1998). The expression of two AOS genes of rice (OsAOS1 and OsAOS2) is activated by mechanical wounding, blast fungus infection, herbivore infestation, and JA treatment (Zeng et al., 2021). In sugarcane, accumulation of ShAOS1 transcripts was reported to increase due to treatments with methyl jasmonate, SA, and abscisic acid (ABA) (Sun et al., 2023).

At present, many AOS genes identified in plant species have been found to play an important role in mediating resistance to fungal pathogens and insects. The functional deficit of mRNA of AOS leads to a decrease in resistance to necrotrophic fungi in Arabidopsis (Chehab et al., 2011), potato (Pajerowska- Mukhtar et al., 2008), onion (Kim et al., 2025), and wheat (Fan et al., 2019). In line with this, the complementation of an Arabidopsis aos knockout mutant with overexpressed gingko HvnAOS1 and HvnAOS2 genes and potato StAOS2 alleles allowed restoring the resistance to Erwinia carotovora and Botrytis cinerea (Pajerowska-Mukhtar et al., 2008; An et al., 2024). Constitutive expression of the ShAOS1 gene, isolated from sugarcane, enhanced the resistance of transgenic Nicotiana benthamiana plants to Fusarium solani (Sun et al., 2023). Similarly, transgenic lines of tobacco overexpressing the onion AOS gene (AcAOS) demonstrated higher resistance to Botrytis squamosa (Kim et al., 2025). In rice, silencing of the OsAOS1 or OsAOS2 genes negatively affected resistance to the chewing herbivore striped stem borer (Chilo suppressalis), resulting in enhanced resistance to the piercing-sucking herbivore brown planthopper (Nilaparvata lugens) (Zeng et al., 2021). An Arabidopsis mutant lacking functional AOS demonstrated increased resistance to the nematode Meloidogyne javanica (Naor et al., 2018).

Changes in the functional activity of AOS genes can have pleiotropic effects on various plant characteristics. Transgenic guayule (Parthenium argentatum) with RNAi-mediated silencing of AOS showed higher rubber content and biomass (Placido et al., 2019), while the disruption of AOS activity in Arabidopsis caused male sterility (Park et al., 2002). Transgenic tobacco overexpressing the wheat AOS gene displayed improved tolerance to excessive zinc stress (Liu et al., 2014). Transgenic emmer wheat plants constitutively expressing the Arabidopsis AtAOS gene showed increased tolerance to osmotic stress (Pigolev et al., 2023), whereas overexpression of the native TaAOS2 gene had a generally negative effect on plant growth and productivity in both emmer and bread wheat (Miroshnichenko et al., 2024a).

In bread wheat (Triticum aestivum L.), the most important cereal crop in temperate climate countries, the AOS family is represented by 12 genes. According to a recent genome-wide analysis, the AOS family consists of four groups of three homoeologous genes located in each sub-genome (A, B, and D) on chromosomes 2, 5, and 6 (Heckmann et al., 2024). The functioning of the twelve AOS-encoded proteins significantly distinguishes the jasmonate system of wheat from that of the model species, Arabidopsis, operated by a single AOS gene. Our data indicate that under non-stress conditions, the total content of jasmonates (12-OPDA, JA, and JA-Ile) in wheat leaves is much lower compared to Arabidopsis (Laudert et al., 1996). Upon mechanical injury of leaves, the level of jasmonates increases in emmer and bread wheat up to 1.5–13 times (Miroshnichenko et al., 2024a, b), whereas in A. thaliana, the content of jasmonates increases tens of times (Kimberlin et al., 2022). In addition, it was reported that the expression of AOS in the leaves of bread wheat could also be induced in response to salts and various pathogenic fungi (Liu et al., 2014; Heckmann et al., 2024). Constitutive overexpression of TaAOS2 resulted in pleiotropic effects in bread wheat, including reduced length of the first four leaves, shortened plant height, and reduced number of seeds collected per spike (Miroshnichenko et al., 2024a). A transgenic seedling of tetraploid emmer wheat overexpressing the AtAOS gene from Arabidopsis showed an increased length of roots and coleoptiles under osmotic stress (Pigolev et al., 2023).

The published data indicate that the knowledge of the functioning of the AOS branch of oxylipin biosynthesis discovered in various species cannot be directly applied to other species. This suggests that the modification of mRNA levels of AOS-encoding genes using reverse genetic techniques (overexpression, RNA interference/silencing, or genome editing) may help to uncover their functional role in specific plant species. To gain new insights into the functions of the AOS-encoding gene family in wheat, the present study aimed to study jasmonate signalling in the case of reduced expression of the TaAOS2 gene, including the modification of hormonal status of transgenic lines and influence of AOS silencing on growth characteristics.

## Materials and methods

Construction of the RNAi expression cassette. The expression cassette pBAR-GFP-AOSi was constructed using two identical fragments of the TaAOS2 gene (TraesCS4A02G061800.1) of 333 bp, which were cloned in opposite orientation to each other and separated by a spacer representing the GUS gene. To avoid non-selective silencing of other genes belonging to the cytochrome P450 family proteins, especially CYP74 enzymes (AOS, hydroperoxide lyase (HPL), and divinyl ether synthase (DES)), we used the fragment outside of the C-domain encoding heme-binding site. The selected harpin arm sequence also did not contain DNA regions, including sequences identical to the HPL and DES genes longer than 12–20 nucleotides. The fragment corresponded to the central part of the protein sequence (from L93 to W203) and demonstrated high nucleotide homology between all AOS genes.

The RNAi construct containing the sequence 3′-TaAOS-5′- GUS-5′-TaAOS-3′ was cloned in two steps. The first RNAi fragment was obtained by PCR from a plasmid containing the full-length AOS2 (TraesCS4A02G061800.1) using primers: 5′-rnaI-short-NotI-GATCGCGGCCGCCAGCT GCTCTTCTCCCTCCTCG and 3′-rnaI-EcoRV-GATCGA TATCCACTTGGCGGCCTTGGTG. This fragment (335 bp) was then cloned into the Gateway pENTR1A dual plasmids at the NotI and EcoRV sites. The second element of the construct, containing GUS and the second RNAi sequence, was obtained by PCR on overlapping DNA templates. The GUS gene sequence was amplified using a pair of primers 5′RNAi-GATGACGGTATCGATAAGCTTGATATCT ACCCGCTT and 3′GUS-(KpnI)-GATCGGTACCATTC GATCGAGTGAAGATCCCTTTCTTG. The second RNAi fragment was amplified with the 5′RNAI-(DraI)-GATCTT TAAATATCCACTTGGCGGCCTTGGTG and 3′RNAi- GATATCAAGCTTATCGATACCGTCATCCTCTTCA CAGGCACCTACATGC primers. After amplification, the two resulting fragments of 933 bp (containing the GUS gene sequence) and 335 bp (containing the RNAi arm sequence) were combined by PCR with common primers (containing restriction sites for DraI and KpnI). After that, the combined GUS-RNAi sequence was cloned into the pENTR1A plasmid, containing the first RNAi fragment, at the restriction sites DraI and KpnI. The resulting construct of inverted TaAOS2 repeats was placed under the control of a strong ubiquitin promoter from maize (ZmUbi1) and an octopine synthase transcription terminator by transfer into the vector pANIC5D (ampR, kanR, bar, pporRFP) using the LR clonase enzyme for Gateway cloning. The completed RNAi cassette (ocsT)- pANIC5D::PZmUbi1-RNAi(3′-TaAOS-5′-GUS-5′-TaAOS- 3′)-Tocs was cut out from pANIC5D at the SacI–SnaBI sites and finally inserted at the SmaI restriction site into the pBARGFP vector for cereal genetic transformation.

Generation of transgenic plants. Transgenic plants of spring bread wheat cv. Chinese Spring were generated using the biolistic-mediated genetic transformation approach as described previously (Miroshnichenko et al., 2024a). After rounds of in vitro selection, the herbicide-resistant rooted plantlets were transferred to the greenhouse, and the resulting mature plants were then analysed by PCR for the introduction of the hairpin construct. Transgenic status of T0 plants was confirmed by the amplification of GFP-specific fragment of 606 bp using primers sGFPFor (5′-GCGACGTAAACGGC CACAAG) and sGFPRev (5′-CCAGCAGGACCATсTGTG ATCG) as described previously (Pigolev et al., 2018). To verify the presence of left and right arms of the RNAi construct, primers TaAOSRi-1 (5′-ATGAACTCGAAGGAGGT GAAGTCGTTG-3′) and panicGUS-1 (5′-CTCTTCAGCG TAAGGGTAATGCGAGGTA-3′) generating a 500 bp product were designed. The integration of the right hairpin arm was confirmed by amplification of a 466 bp fragment using the TaAOSRi-1 (5′-ATGAACTCGAAGGAGGTGAAGTC GTTG-3′) and panicGUS-2 (5′-CTGCACTCAATGTACA CCGACATGTG-3′) primers. T1 seed progeny resulting from self-pollinated primary T0 plants were screened for GFP fluorescence of pollen to identify homozygous transgenic T1 sub-lines stably inheriting the foreign insertion, as previously described (Pigolev et al., 2018). Sets of T3–T4 seeds of four discovered homozygous sub-lines were used for further analysis.

TaAOS2 expression analysis. Extraction of total RNA from wheat leaf tissue, subsequent cDNA synthesis, and quantitative real-time RT-PCR analysis were performed as described previously (Pigolev et al., 2023). To detect changes in the expression levels of the TaAOS2 gene, the forward primer TaAOSshbF (5′-GGCCGGAGAGAAGTTCCAC-3′) and the reverse primer TaAOSshbR (5′-CTTCTCCAGCGCCTC TATCG-3′) were used. Transcript levels were quantified with QuantStudio™ 5 Real-Time PCR Cycler (Thermo Fisher Scientific) using TaWIN1 as a reference gene.

JA, JA-Ile, SA and ABA analysis. Intact and mechanically damaged leaves of non-transgenic plants and mechanically damaged leaves of four transgenic lines were used for phytohormone analysis. To induce abiotic stress, blades of fully opened 3rd leaves were wounded with forceps as described earlier (Pigolev et al., 2023). 30 min after mechanical injury, the leaves were collected and frozen in liquid nitrogen. The intact leaves were sampled in parallel with wounded tissues. For phytohormone analysis, the leaf tissues were ground in liquid nitrogen with a mortar and pestle. Extraction of hormones was performed according to the previously described procedure (Degtyaryov et al., 2023). Dihydrojasmonic acid (Merck KGaA, Darmstadt, Germany) and deuterated salicylic acid (Cayman Chemical, USA) were used as internal standards. Extracted samples were treated with trimethylsilyldiazomethane to produce methyl ester derivatives, which were analyzed on a gas chromatograph coupled with a mass spectrometer detector Chromatec-Crystal 5000 (Chromatec, Yoshkar-Ola, Russia) operating in electron ionization mode. One-microliter samples were injected by autosampler in splitless mode at 250 °C, and separated on a CR-5MS column (length 30 m, inner diameter 0.25 mm, film thickness 0.25 μm) with helium being used as a carrier gas (constant flow 0.7 ml/ min). Oven temperature programming was as follows: hold at 40 °C for 1 min after injection, ramp at 15 °C/min to 150 °C, then increase at 10 °C/ min to 250 °C, hold for 10 min. Mass spectral analysis was done in selective ion monitoring mode (SIM). The fragment ions of esterified forms of phytohormones monitored were as follows: jasmonic acid (JA) 224, jasmonoil-isoleucine conjugate (JA-Ile) 146, dihydro-JA 83, salicylic acid (SA) 152, abscisic acid (ABA) 190, and deuterated SA derivative SA-d4 156. The Chromatec Analytic 3 program was used for the data analysis.

Plant growth and productivity analysis. For the analysis, two homozygous transgenic lines were cultivated together with non-transgenic control in a greenhouse. Two plants were grown in one-liter pot using a 16 h day/8 h night regime. Light intensity was up to 200 μmol·m−2s−1. The day temperature was 25 ± 2 °C; at night, the temperature was 20 ± 2 °C. A minimum of 15 pots per line were cultivated. The growth parameters analyzed were as follows: the length of the 1st, 2nd, 3rd, and 4th leaves (twenty leaves were measured), the height of plants (15 pots were analyzed), and the date of the anthesis (30 plants were analyzed). The average number of seeds per spike and the weight of 1,000 seeds were recorded at the end of cultivation. Statistically significant differences between control and transgenic lines were confirmed by Student’s t-test.

## Results

**Generation of transgenic wheat lines with silenced TaAOS2 expression**

**Fig. 1.**
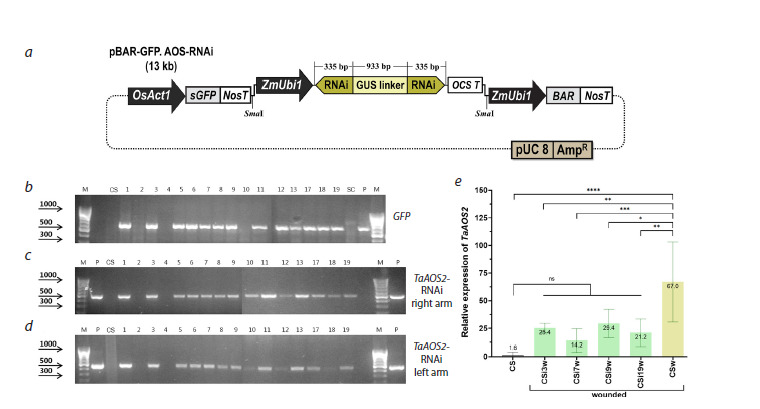
Generation, selection, and analysis of transgenic wheat plants expressing a construct for silencing of the TaAOS2 gene. a, Schematic diagram of the pBAR-GFP-AOSi vector used for the transformation of bread wheat cv. Chinese Spring designed for the induction of RNAi-mediated silencing of the TaAOS2 gene. b–d, Example of PCR analysis of primary putative transgenic plants for the insertion of the heterologous sequences. b, Amplification of the GFP gene fragment; c, d, amplification of the right (c) and left (d) arms of the hairpin sequence for TaAOS2 silencing; lane M, DNA ladder as a molecular weight marker; lane P, DNA of pBAR-GFP-AOSi; lane CS, non-transgenic wheat plant Chinese Spring; lanes labelled 1–19 represent putative transgenic wheat plants established in the greenhouse. e, Relative expression of the TaAOS2 gene in intact and wounded leaves of non-transgenic plants and wounded transgenic plants. Stars above the graphs indicate statistically significant differences according to Student’s t-test: * p ≤ 0.05, ** p ≤ 0.01, *** p ≤ 0.005, **** p ≤ 0.001, ns, non-significant.

To produce transgenic plants, 630 morphogenic calli of wheat cv. Chinese Spring were transformed using a particle inflow gun with the pBAR-GFP-AOSi plasmid, carrying the hairpin loop sequence for silencing the TaAOS2 gene (Fig. 1a). As a result of the transformation, 43 putative transgenic plantlets were regenerated using a dual selection approach (GFP+BAR) and adapted to ex vitro greenhouse conditions. The efficiency of transgenic plant production was 5.6 %, as PCR analysis detected amplification of a fragment of the GFP reporter gene in the DNA extracts of 34 primary independent wheat plants (Fig. 1b). The insertion of the complete RNAi sequence (3→5′-TaAOS-GUS-5′→3′-TaAOS) was confirmed for 33 independent T0 plants by amplification of both the left and the right arm fragments using specific primers (Fig. 1c, d). In order to get a homozygous transgenic population, T1 progenies from T0 plants were analysed for segregation of the introduced construct by detection of GFP expression in pollen plants and in T2 embryos. T1 progenies of several transgenic lines showed the segregation for GFP expression that fit the Mendelian 3:1 ratio for a single dominant locus. T3 seeds from homozygous T2 individuals of primary transgenic plants CSi3, CSi7, CSi9, and CSi19 were used for further analysis.

To identify RNAi-mediated silencing, we used wounded leaves of transgenic lines CSi3, CSi7, CSi9, and CSi19 and compared the TaAOS2 expression in these samples with intact and damaged leaves of non-transgenic plants. Quantitative real-time PCR showed that mechanical damage to non-transgenic leaves increased the level of TaAOS2 mRNA accumulation by about 40 times from 1.6 to 67.0 (Fig. 1e). In wounded transgenic lines, TaAOS2 expression was 2.3–4.7 times lower compared to the damaged leaves of non-transgenic control, whereas the difference with intact non-transgenic control was not statistically confirmed.

**Effect of TaAOS2 silencing on the accumulation of JA, JA-Ile, ABA, and SA in leaves of transgenic wheat plants**

**Fig. 2.**
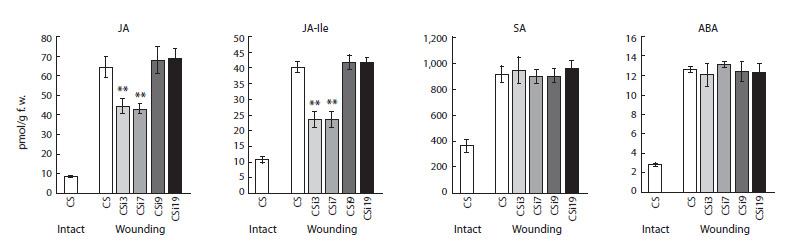
The content of jasmonic acid (JA), jasmonoyl-isoleucine conjugate (JA-Ile), salicylic acid (SA), and abscisic acid (ABA) in intact and wounded leaf tissues of non-transgenic wheat (Chinese Spring, CS) and wounded transgenic lines with silencing of TaAOS2 (CSi3, CSi7, CSi9, and CSi19). Stars indicate a statistically significant difference from the wounded non-transgenic Chinese Spring leaves at p ≤ 0.01, as assessed using Student’s t-test.

The basal levels of jasmonic acid (JA) and jasmonoyl-isoleucine conjugate (JA-Ile) in the intact leaves of Chinese Spring were barely detectable by (GC)–MS chromatography and fluctuated around 10 pmol/g f. w. (Fig. 2). When the leaves of non-transgenic plants were subjected to mechanical wounding, the level of JA and JA-Ile accumulation increased to 64.4 and 40.3 pmol/g f. w., correspondingly. Endogenous concentrations of jasmonates in injured leaves of transgenic plants differed among the four analyzed transgenic lines and did not correlate with the level of TaAOS2 expression. Two RNAi lines, CSi3 and SCi7, contained only half of the amount of JA-Ile compared to non-transgenic Chinese Spring plants subjected to wounding. Two other transgenic lines, CSi9 and SCi19, showed no difference in JA-Ile content. Measurement of the JA level revealed a similar pattern: JA production in wounded leaves of transgenic lines CSi9 and SCi19 (67.8–68.8 pmol/g f. w.) did not differ from the non-transgenic wounded plants; JA content in transgenic lines CSi3 and SCi7 decreased to 43–44 pmol/g f. w. ( p < 0.05).

After wound treatment of non-transgenic wheat plants, a significant increase in production of salicylic acid (SA) and abscisic acid (ABA) was observed (Fig. 2): the level of SA increased around 2.5 times from 362.7 to 914.2 pmol/g f. w., wounding stimulated a 4-fold increase of ABA accumulation in leaves (2.9 vs. 12.6 pmol/g f. w.). Levels of both phytohormones (SA and ABA) were not significantly different in wounded transgenic lines and non-transgenic control.

**Analysis of plant growth and productivity of RNAi transgenic lines with modified content of jasmonates**

**Fig. 3.**
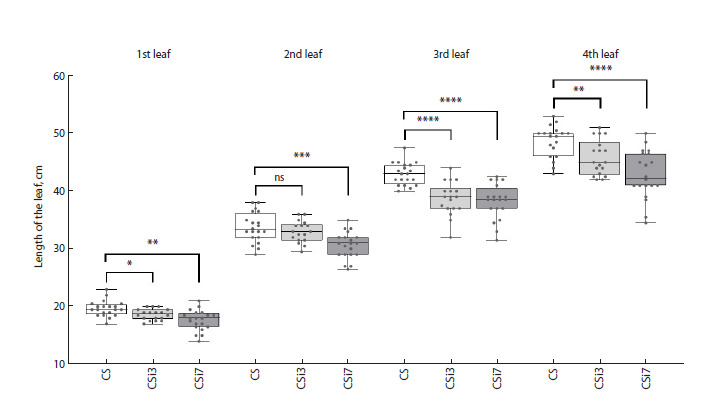
Analysis of leaf length of non-transgenic bread wheat Chinese Spring and transgenic lines with silencing of TaAOS2 (CSi3 and CSi7). Values represent the lengths of 1st, 2nd, 3rd, and 4th leaves measured in 18–20 plants; the line represents the median value, the box margins are SD of the mean, and the bar margins reflect the distribution of values from min to max. Stars indicate statistically significant differences calculated according to Student’s t-test: * p < 0.05, ** p < 0.01, *** p < 0.005, **** p < 0.001, ns, non-significant.

In this experiment, two transgenic RNAi lines, CSi3 and SCi7, which showed suppression of TaAOS2 gene activity resulting in altered JA and JA-Ile content, were compared with the non-transgenic parent variety Chinese Spring. The measurement of the length of four first fully developed leaves of greenhouse-grown plants revealed a decrease in leaf length in both RNAi transgenic lines (Fig. 3). In AOS silencing line CSi7, the changes were more pronounced, as the average lengths of the 1st, 2nd, 3rd, and 4th leaves were 2.2 cm ( p < 0.01), 3.1 cm ( p < 0.005), 4.8 cm ( p < 0.001), and 5.8 cm ( p < 0.001) shorter, correspondingly, compared to the non-transgenic parent plants. The leaves of the transgenic CSi3 line were shorter by 2.5–10 %, and significant leaf length changes were confirmed by Student’s t-test for 1st, 3rd, and 4th leaves (at p < 0.05 to p < 0.001).

**Fig. 4.**
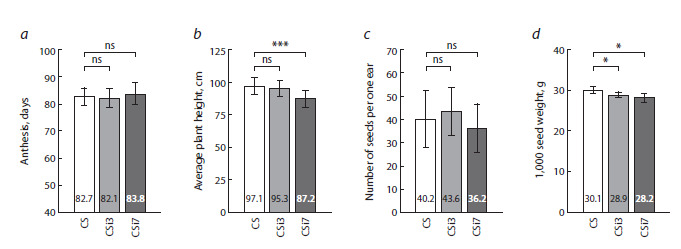
Analysis of plant growth and productivity of RNAi transgenic wheat lines with modified content of jasmonates. a, Anthesis date of the first ear; b, average plant height; c, mean number of seeds per ear; d, weight of 1,000 seeds. Stars indicate statistically significant differences of transgenic RNAi lines with silencing of TaAOS2 (CSi3 and CSi7) from non-transgenic Chinese Spring (SC) plants calculated according to Student’s t-test: * p &0.05, *** p & 0.005, ns, non-significant

In addition to a reduction in length of leaves, the transgenic CSi7 line also showed significant changes in plant development. On average, CSi7 plants were 9.9 cm shorter than nontransgenic control, while the transgenic CSi3 line showed almost the same plant height as non-transgenic Chinese Spring plants (Fig. 4b).

The average time of the first spike appearance and anthesis was similar in transgenic and non-transgenic plants. A minor change in the average date of flowering observed in RNAi line CSi7 (84 days vs. 83 days in CS) was not proved to be statistically significant (Fig. 4a).

Analysis of productivity revealed a variation in the number of seeds collected from one ear of RNAi lines. Compared to control plants (40.2 seeds/ear), the average number of seeds collected from CSi3 plants was slightly higher (43.6 seeds/ ear), while in CSi7 plants, it was slightly reduced (36.2 seeds/ ear). Statistical analysis, however, did not confirm the differences between the non-transgenic control and RNAi transgenic lines to be significant (Fig. 4c).

The measurement of grain weight revealed a decrease in the average weight of 1,000 seeds by 4–7 % (p < 0.01) in both transgenic RNAi lines (Fig. 4d). The mean values for 1,000 grain weight were reduced from 30.1 g in non-transgenic control plants to 28.9 and 28.2 g in the SCi3 and CSi7 lines, correspondingly.

## Discussion

Jasmonates are known as stress-related plant phytohormones, the activity of which is stimulated by various external stressors. Last decade, to study the functioning of jasmonates in plants, reverse genetics methods have been actively used to regulate the expression of specific genes of the jasmonates biosynthesis pathway. In our previous experiments, the functions of AOS genes in two wheat species were studied using the overexpression strategy (Pigolev et al., 2023; Miroshnichenko et al., 2024a). The present study focused on the generation and initial characterization of transgenic bread wheat plants in which the endogenous AOS gene activity was suppressed by RNA interference.

Previously, producing transgenic lines overexpressing the AOS gene was challenging due to the low output of transgenic plants in both emmer and bread wheat (Miroshnichenko et al.,2024a). It was observed that independently of the AOS gene variant used for the design of the overexpression cassette (TaAOS2 from bread wheat or AtAOS from Arabidopsis), morphogenic tissues were characterized by rapid aging and massive necrosis after the biolistic delivery. This resulted in an extremely low rate of transgenic plants production, amounting to 0.3–1.0 % for bread wheat (cv. Saratovskaya 60) and 0.5–1.7 % for emmer (cv. Runo), which was 5–10 times less in comparison with genetic transformation by an empty vector (Miroshnichenko et al., 2024a). In the present study, the down-regulation of AOS had no negative effect on the morphogenetic development of wheat tissue culture, and the production of transgenic wheat plants by introducing an RNA interference cassette was not associated with any difficulties. Transgenic plantlets were readily produced from GFP-positive wheat tissues of cv. Chinese Spring achieving the final genetic transformation efficiency of 5.2 % for the hairpin insertions. The resulting transformation rate is significantly higher compared to our previous experiments involving the other pGFP-BAR-based vector (Miroshnichenko et al., 2022), and it is equal to or even higher than the transformation efficiency in other publications, describing biolistic-mediated transformation of Chinese Spring cultivar (Takumi, Shimada, 1997; Harvey et al., 1999).

Overexpression and silencing of AOS by the genetic transformation of wheat are supposed to have opposite effects on the capacity of cells to accumulate jasmonates. As it is shown in the present study, the level of TaAOS2 activity in transgenic wheat tissues expressing the RNAi construct remains low under the induced stress (wounding) and results in weaker accumulation of JA and JA-Ile content (Fig. 2). We suppose that wheat explants transiently expressing the RNAi construct also accumulate less jasmonates when subjected to chemical stress during the selection of transgenic plants on herbicide-containing medium. Considering the previously reported increase of the jasmonate content in transgenic wheat tissues under stress conditions due to the strong constitutive activity of AOS, especially in case of TaAOS2 overproduction (Miroshnichenko et al., 2024a), these findings, taken together, allow suggesting that high TaAOS2 expression and increased JA content are negative regulators of morphogenesis and somatic embryogenesis in wheat tissue culture, as it was demonstrated for other species (Kamińska, 2021).

The data obtained in the present study suggest that the abiotic stress (wounding) applied to wheat plants of bread wheat cv. Chinese Spring promotes the expression of the TaAOS2 gene and causes higher production and accumulation of various phytohormones, including JA, JA-Ile, SA, and ABA. The result supports previous studies, where the same stimulus induced a similar response in plants of another bread wheat cultivar, Saratovskaya 60, as well as in tetraploid emmer wheat cv. Runo (Pigolev et al., 2023; Miroshnichenko et al., 2024a, b). The obtained data are consistent with data obtained for other plant species: the wound response in wheat is similar to that in rice (Zeng et al., 2021), but significantly weaker than in Arabidopsis (Kimberlin et al., 2022) and potato (Pajerowska-Mukhtar et al., 2008). Previously, transgenic guayule lines showed increased SA content due to silencing of the AOS gene (Placido et al., 2019); in contrast to guayule RNAi lines, there was no alteration in SA content in AOSsilenced wheat lines. Such results support the idea that the effect of modified AOS activity is highly dependent on the plant species and functional specialization of AOS genes. In line with this, in guayule, which is a dicotyledonous plant species, the silencing of AOS was easily detectable in intact plants. In another dicot species, potato, downregulation of StAOS1 was also not detected in undamaged leaves due to extremely low mRNA levels, while the silencing of the StAOS2 gene was easily detected as intact dsRNAi transgenic lines accumulated no more than 10 % of the StAOS2 transcript compared to control plants (Pajerowska-Mukhtar et al., 2008).

Due to the limited number of publications on silencing of AOS genes in cereal species (Naor et al., 2018; Fan et al., 2019), information on plant growth and productivity is lacking. In the present study, the AOS-silenced wheat lines showed a tendency to grow shorter leaves at the juvenile stage and demonstrated a decrease in final plant height. Surprisingly, a similar trend was observed in both transgenic emmer and bread wheat overexpressing TaAOS2. However, despite the growth modification, wheat lines with the knockdown of TaAOS2 expression showed no change in the mean flowering date. This result is contrasting to a previous study demonstrating that AOS-silenced guayule plants showed higher biomass accumulation, enhanced stem branching, increased photosynthetic rate, and increased rubber content (Placido et al., 2019). This increase was associated with elevated rubber transferase enzyme activity, increased SA content, and decreased ABA levels (Placido et al., 2019). In contrast to RNAi lines of guayule, AOS-silenced wheat lines showed no significant changes in endogenous SA and ABA accumulation, while similarly to guayule, JA and JA-Ile contents were also reduced, explaining the markedly different effects between the two species. The observed differences can be associated with the presence of a larger number of AOS genes in hexaploid wheat, which can perform compensatory functions during TaAOS2 silencing or with other differences in the complex regulatory system responsible for maintaining the intracellular level of jasmonates and/or jasmonate signaling. This question certainly requires further study.

The downregulation of AOS activity did not affect the fertility of transgenic wheat plants. For comparison, the complete disruption of AOS function in the aos mutant of Arabidopsis was reported to induce male sterility and failure of seed formation (Park et al., 2002). In the present study, wheat RNAi lines showed normal seed set, probably because there was no complete silencing of TaAOS2. This observation is consistent with the previous publications on RNAi-mediated silencing of AOS genes in wheat, rice, and guayule, where transgenic homozygous progenies were successfully generated by self-pollination of primary plants (Naor et al., 2018; Fan et al., 2019; Placido et al., 2019). The productivity analysis performed in the present study revealed that the grain weight of RNAi lines tended to decrease, while the mean number of seeds per one ear was not changed. Conversely, when we previously overexpressed the TaAOS2 gene in bread wheat cv. Saratovskaya 60, this caused a decrease in the seed number per ear (Miroshnichenko et al., 2024a).

## Conclusion

Based on these experimental results, we can assume that the downregulation of TaAOS2 had no clear beneficial effect on the growth and productivity of bread wheat. However, these results were obtained on plants grown without any temporal or prolonged biotic or abiotic stresses. Given that the functional activity of AOS in wheat is highly dependent on external stimuli, our further research will focus on the analysis of RNAi lines subjected to pathogen attacks and temperature stress. This will help to expand the understanding of the functional effects of AOS suppression in wheat and may provide more information on the possibility of precise genetic regulation of the jasmonate biosynthetic pathway to achieve better growth and productivity under various stress conditions

## Conflict of interest

The authors declare no conflict of interest.
